# Sodium butyrate potentiates insulin secretion from rat islets at the expense of compromised expression of β cell identity genes

**DOI:** 10.1038/s41419-022-04517-1

**Published:** 2022-01-19

**Authors:** Shushu Wang, Miaomiao Yuan, Linlin Zhang, Kecheng Zhu, Chunxiang Sheng, Feiye Zhou, Zhaoqian Xu, Qianqian Liu, Yun Liu, Jieli Lu, Xiao Wang, Libin Zhou

**Affiliations:** 1 Department of Endocrine and Metabolic Diseases, Shanghai Institute of Endocrine and Metabolic Diseases, Ruijin Hospital, Shanghai Jiao Tong University School of Medicine, Shanghai, China; 2 Shanghai National Clinical Research Center for Metabolic Diseases, Key Laboratory for Endocrine and Metabolic Diseases of the National Health Commission of the PR China, Shanghai National Center for Translational Medicine, Ruijin Hospital, Shanghai Jiao Tong University School of Medicine, Shanghai, China

**Keywords:** Transcriptomics, Endocrine system and metabolic diseases

## Abstract

Short-chain fatty acids (SCFAs) produced by the gut microbiota have been well demonstrated to improve metabolic homeostasis. However, the role of SCFAs in islet function remains controversial. In the present study, none of the sodium acetate, sodium propionate, and sodium butyrate (SB) displayed acute impacts on insulin secretion from rat islets, whereas long-term incubation of the three SCFAs significantly potentiated pancreatic β cell function. RNA sequencing (RNA-seq) revealed an unusual transcriptome change in SB-treated rat islets, with the downregulation of insulin secretion pathway and β cell identity genes, including Pdx1, MafA, NeuroD1, Gck, and Slc2a2. But these β cell identity genes were not governed by the pan-HDAC inhibitor trichostatin A. Overlapping analysis of H3K27Ac ChIP-seq and RNA-seq showed that the inhibitory effect of SB on the expression of multiple β cell identity genes was independent of H3K27Ac. SB treatment increased basal oxygen consumption rate (OCR), but attenuated glucose-stimulated OCR in rat islets, without altering the expressions of genes involved in glycolysis and tricarboxylic acid cycle. SB reduced the expression of Kcnj11 (encoding K_ATP_ channel) and elevated basal intracellular calcium concentration. On the other hand, SB elicited insulin gene expression in rat islets through increasing H3K18bu occupation in its promoter, without stimulating CREB phosphorylation. These findings indicate that SB potentiates islet function as a lipid molecule at the expense of compromised expression of islet β cell identity genes.

## Introduction

The pancreatic β cell plays a vital role in the maintenance of glucose homeostasis by secreting an appropriate amount of insulin [1]. Pancreatic β cell dysfunction is one of the main contributors to the initiation and progression of type 2 diabetes [2]. To respond with an appropriate insulin release to fluctuating glucose levels, it is indispensable for β cells to develop a maturation machinery that defines their functional identity. Intensive studies in animal models have led to the identification of multiple molecular markers which maintain the function and identity of β cells, including Slc2a2, Gck, Pdx1, MafA, Nkx2-2, Nkx6-1, and NeuroD1 [3–7]. The down-regulation of these important functional genes and transcription factors indicates the loss of mature β cell identity. Protein acetylation is involved in the maintenance of β cell function and identity. H3K27ac has been considered a key marker of cell identity [8]. The loss of β cell identity under stressful environments such as glucotoxicity is usually accompanied by β cell dedifferentiation towards progenitor-like cells or transdifferentiation to other islet cell types [3, 9].

As an energy sensor, the β cell adapts its response to variations in the level of multiple circulating nutrients including glucose, amino acids, and free fatty acids (FFAs), showing great plasticity in insulin secretion [10]. Glucose-stimulated insulin secretion (GSIS) is completely ablated in lipid-depleted islets, whereas restoring FFAs rescues insulin secretion, indicating an important role of FFAs in modulating β cell function [11, 12]. Fatty acids are grouped into long- (C12-C22), medium- (C7-C12), and short-chain fatty acids (SCFAs, C2-C6) according to their carbon chain length [13]. The insulinotropic effect of FFAs is profoundly influenced by chain length and degree of saturation [14].

SCFAs could be produced by the intestinal microbiota or by catabolism of long-chain fatty acids or branched-chain amino acids [15–17]. The three most common SCFAs, acetate, propionate, and butyrate have been demonstrated to increase insulin sensitivity, induce weight loss, reduce inflammation, etc [18]. However, no clear consensus has been achieved about the effect of SCFAs on β cell function. Some studies reported that acetate, propionate, and butyrate potentiated GSIS [19–22], while others displayed a contrary result or no effects [23–25]. Besides as nutrient substrates, SCFAs act as signaling activators for the G-protein-coupled-receptors as well as histone deacetylase (HDAC) inhibitors [26–28]. However, the mechanism underlying SCFAs-regulated β cell function remains uncertain.

In this current study, long-term incubation of three SCFAs sodium acetate (SA), sodium propionate (SP), or sodium butyrate (SB) significantly amplified insulin secretion from rat islets in response to glucose and amino acid. Unexpectedly, RNA sequencing (RNA-seq) revealed that SB downregulated multiple key transcription factors and functional genes of β cells. The insulinotropic function of SB was attributed to increased insulin gene expression mediated by H3K18bu in its promoter region and elevated intracellular calcium concentration ([Ca^2+^]_i_) by ablating K_ATP_ channels.

## Results

### SCFAs potentiates β cell function while downregulating insulin secretion pathway

**Fig. 1:**
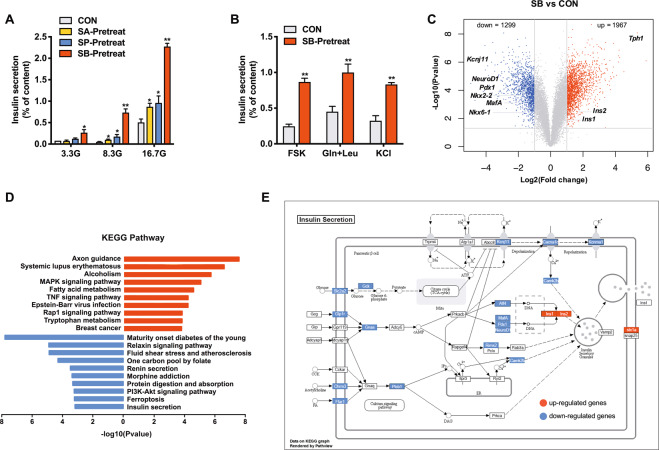
Effects of short-chain fatty acids on insulin secretion and transcriptome in rat islets. **A** Rat islets were pretreated with 5 mM sodium acetate (SA), 5 mM sodium propionate (SP), and 5 mM sodium butyrate (SB) at 3.3 mM glucose for 24 h, and then stimulated with 3.3, 8.3, or 16.7 mM glucose for 1 h. Insulin secretion was measured. **B** Rat islets were pretreated with 5 mM SB at 3.3 mM glucose for 24 h, then stimulated with 10 μM forskolin (FSK), 5 mM glutamine (Gln) and leucine (Leu), and 35 mM KCl for 1 h, and insulin secretion was measured. **C** Volcano plot of differentially expressed genes from RNA-seq analysis of isolated rat islets treated with or without 5 mM SB for 24 h (*n* = 3). The thresholds set for upregulated (red) and downregulated (blue) genes were a fold change ≥ 2.0 and a *p-value* ≤ 0.05. **D** KEGG pathway analysis of upregulated (red) and downregulated (blue) genes. **E** Differentially expressed genes in the insulin secretion KEGG pathway in SB-treated islets compared to the control islets. Green indicates downregulation (fold change ≤ 0.5) and yellow indicates upregulation (fold change ≥ 2). Gray label marks genes with non-significant changes. Data were given as mean ± SD for three separate experiments. *p < 0.05 and **p < 0.01 *vs* control (CON) group.

To determine the impact of SCFAs on islet β cell function, isolated rat islets were treated with SA, SP, and SB. Consistent with previous reports [27], SCFAs were without acute effect on basal and glucose-induced insulin secretion (Fig. S1A). When the incubation time was prolonged to 24 h, SCFAs significantly increased insulin secretion from rat islets at 3.3, 8.3, and 16.7 mM glucose (Fig. 1A), without changing insulin content (Fig. S1B). At the concentration of 5 mM, SCFAs displayed no cytotoxicity on INS-1 cells (Fig. S1C). In addition, long-term treatment of SB further enhanced insulin secretion in the presence of cAMP-elevating agent forskolin (FSK), leucine and glutamine, and high potassium (Fig. 1B), without acute effects under the same conditions (Fig. S1D).

To understand the molecular basis underlying the insulinotropic action of SCFAs, the global gene expression patterns were analyzed by RNA-seq in rat islets incubated with or without SB for 24 h. 3266 differentially expressed genes (1967 upregulated and 1299 downregulated genes) were identified between SB group and control group (Fig. 1C) and subjected to KEGG pathway analysis. Interestingly, maturity-onset diabetes of the young (MODY) pathway including a series of β cell key transcription factors and functional genes, was one of the most significantly downregulated pathways. The insulin secretion pathway was included in the top ten enriched KEGG pathways downregulated by SB (Fig. 1D), in contradiction with its insulinotropic effect. The key differentially expressed genes involved in insulin secretion from glucose metabolism to ion channel and insulin gene regulation were marked in Fig. 1E.

### SB inhibits expressions of multiple β cell identity genes

**Fig. 2:**
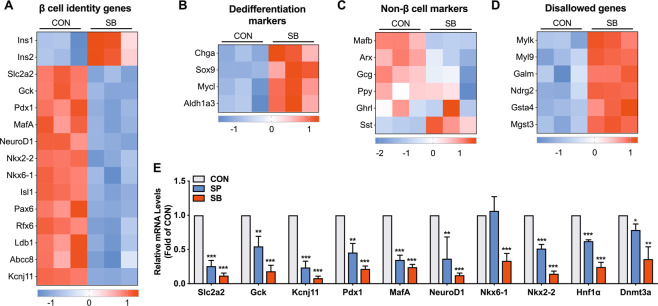
Effects of sodium butyrate on the expression of β cell identity genes. **A** Heatmap of β cell identity genes in control and sodium butyrate (SB)-treated rat islets. **B** Heatmap of dedifferentiation markers. **C** Heatmap of non-β cell markers. **D** Heatmap of disallowed genes. **E** RT-qPCR analysis of β cell identity genes in rat islets incubated with 5 mM SP or 5 mM sodium propionate (SB) for 24 h. Data were given as mean ± SD for three separate experiments. *p < 0.05, **p < 0.01, and ***p < 0.001 *vs* control (CON) group.

We further presented a comprehensive assessment of the effects of SCFAs on the expression of genes related to β cell functional maturity. The expressions of key β cell functional genes Slc2a2, Gck, and Kcnj11, as well as key regulators of β cell identity including Pdx1, MafA, Nkx2-2, Nkx6-1, and NeuroD1, were considerably decreased by SB treatment, while the expressions of insulin genes (Ins1 and Ins2) were increased (Fig. 2A). Similar effects of SB were observed in mouse islets (Fig. S2A). Furthermore, the expressions of Pdx1, MafA, NeuroD1, Slc2a2, Gck, and Kcnj11 were derepressed in INS-1 cells and mouse islets after SB was withdrawn, suggesting that the inhibitory effects of SB were reversible (Fig. S2B, C). Given the previous reports [3, 9] that the loss of β cell identity was usually accompanied with β cell dedifferentiation and transdifferentiation, we then investigated the markers of the two processes. Aldh1a3, Chga, Sox9, and Mycl mRNA expressions were increased in SB-treated islets (Fig. 2B). Except for Sst, no other islet hormone genes such as Gcg, Ppy, and Ghrl exhibited significant increases in rat islets treated with SB (Fig. 2C).

The repression of disallowed genes is also crucial for proper β cell mature phenotype [29]. Among 47 disallowed genes identified by Pullen et al. [30] and Thorrez et al. [31], six genes were upregulated in SB-treated islets (Fig. 2D). It has been reported that DNA and histone methylation is involved in the repression of disallowed genes [32]. Interestingly, the protein level of DNA methyltransferase 3a (Dnmt3a) displayed a significant decrease in SB-treated islets (Fig. S2D). Similar to the action of SB, SP also exerted inhibitory effects on the expression of β cell identity genes in rat islets (Fig. 2E).

### Role of H3K27Ac in SB-modulated expression of β cell identity genes

**Fig. 3:**
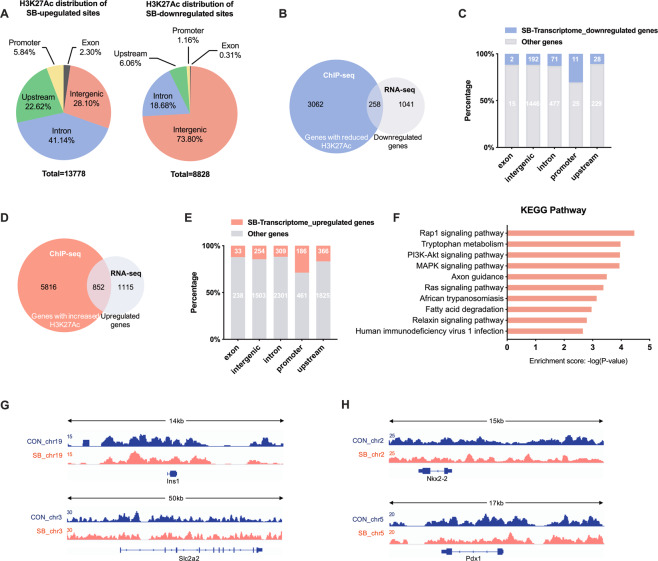
Role of H3K27Ac in sodium butyrate-induced changes of gene expressions shown by ChIP-seq analysis. **A** Genomic distribution of H3K27Ac enriched peaks regulated by sodium butyrate (SB) (fold change > 2.0, *p*-value < 0.001). **B** Venn diagram of the overlap between annotated genes with downregulated H3K27Ac occupation and downregulated genes identified in RNA-seq. **C** Genomic distribution of both H3K27Ac enriched peaks and corresponding genes downregulated by SB (blue). **D** Venn diagram of the overlap between annotated genes with upregulated H3K27Ac occupation and upregulated genes identified in RNA-seq. **E** Genomic distribution of both H3K27Ac enriched peaks and corresponding genes upregulated by SB (orange). **F** KEGG pathway analysis on the overlapping upregulated genes by SB at mRNA level and H3K27Ac level in their promoter. **G**, **H** Detailed H3K27Ac patterns of Ins1, Slc2a2, Nkx2-2, and Pdx1 from ChIP-Seq. Gene structure and chromosomal location are shown.

H3K27Ac has been regarded as a biomarker of cell type-specific promoters and enhancers involved in key cell identity gene expressions [8]. Therefore, we performed H3K27Ac ChIP-sequencing (ChIP-seq) analysis in SB-treated rat islets. A total of 13778 upregulated and 8828 downregulated H3K27Ac peaks were identified. Most upregulated or downregulated H3K27Ac peaks were located in the intergenic and intron region (Fig. 3A). We further conducted an overlap analysis on H3K27Ac ChIP-seq data with RNA-seq data of SB-treated rat islets. As shown in Fig. 3B, ChIP-seq identified 3320 genes with reduced H3K27Ac peaks in one or more genetic regions, of which 258 genes were downregulated by SB at the mRNA level. Among 36 genes with hypoacetylation of H3K27 in their promoters, 11 genes exhibited decreased mRNA levels, with the highest proportion than those in other regions (Fig. 3C). In addition, 6668 genes were identified with elevated H3K27Ac level, of which 852 genes were upregulated by SB at the mRNA level (Fig. 3D). Among 647 genes with elevated H3K27Ac level in their promoters, there were 186 genes upregulated by SB, showing the highest proportion than those in other regions (Fig. 3E). These results indicate a strong correlation of H3K27Ac level in promoter with gene transcription. We further performed KEGG pathway analysis on the overlapping 186 genes. Fatty acid degradation pathway and tryptophan metabolism pathway were significantly enriched (Fig. 3F). However, the level of H3K27Ac showed no significant changes in the promoter regions of Ins1, Slc2a2, Pdx1, and Nkx2-2 after SB treatment (Fig. 3G, H). These findings suggest that other alternative mechanisms are involved in SB-mediated expressions of β cell identity genes.

### SB represses β cell identity genes independent of its deacetylase activity

**Fig. 4:**
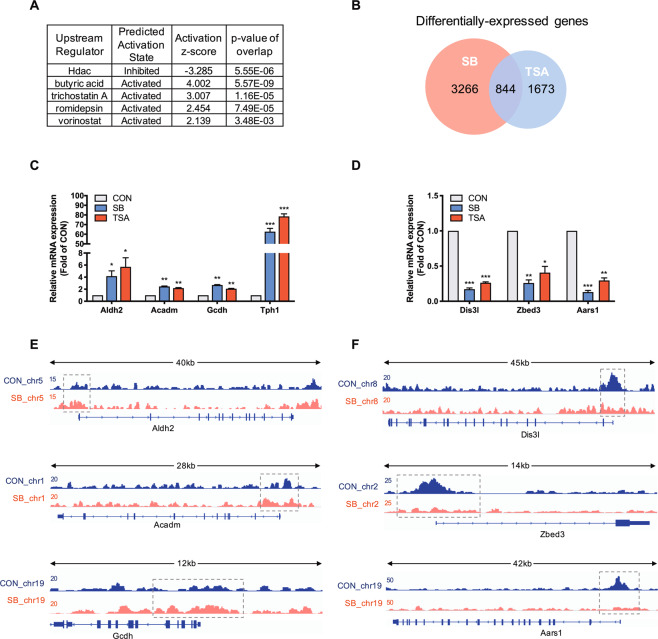
Comparative analysis of the gene expression profiles from sodium butyrate- and trichostatin A-treated rat islets. **A** Upstream regulator analysis of Ingenuity Pathway Analysis (IPA) on sodium butyrate (SB)-regulated genes. **B** Overlapping differentially expressed genes in rat islets regulated by both SB and trichostatin A (TSA). **C**, **D** RT-qPCR analysis of commonly upregulated and downregulated genes in rat islets by 5 mM SB and 200 nM TSA. **E** H3K27Ac patterns of Aldh2, Acadm, and Gcdh from ChIP-seq. Gene structure and chromosomal location are shown, with the gray dotted box highlighting the upregulated peaks in their promoter regions by SB. **F** H3K27Ac patterns of Dis3l, Zbed3, and Aars1 from ChIP-Seq. Gene structure and chromosomal location are shown, with the gray dotted box highlighting the downregulated peaks in their promoter regions by SB. Data were given as mean ± SD for three separate experiments. *p < 0.05, **p < 0.01, ***p < 0.001 *vs* control (CON) group.

To further explore the molecular mechanism of SB, the prediction of upstream regulator was performed using Ingenuity Pathway Analysis (IPA) based on all differentially expressed genes. HDAC was predicted to be inhibited while four HDAC inhibitors butyric acid, trichostatin A (TSA), romidepsin, and vorinostat were predicted to be activated (Fig. 4A), suggesting involvement of acetylation in SB-mediated changes of β cell transcriptome. Like two HDAC inhibitors MS-275 and TSA, three SCFAs significantly increased the acetylation levels of histone H3 and H2 in INS-1 cells (Fig. S2E). We performed an overlapping analysis on two sets of gene expression profiles between SB- and TSA-treated rat islets. There were 844 differentially expressed genes consistently regulated by SB and TSA (Fig. 4B). Among these genes, Aldh2, Acadm, Gcdh, and Tph1 were upregulated by both SB and TSA (Fig. 4C) while Dis3l, Zbed3, and Aars1 were downregulated by the two agents (Fig. 4D). H3K27Ac levels in the promoters of these genes displayed a corresponding change in the presence of SB except for Tph1 (Fig. 4E, F), suggesting involvement of acetylation in the regulation of gene expression. However, unlike in SB-treated islets, the key β cell functional genes and identity genes such as Slc2a2, Gck, Pdx1, NeuroD1, Nkx6-1, Ins1, and Ins2 showed no significant changes in TSA-treated islets except for Kcnj11 (Fig. S3). These results further indicate that there exists a non-acetylation mechanism involved in SB-mediated regulation of islet β cell identity genes.

### SB-potentiated insulin secretion is independent of glucose oxidation in rat islets

**Fig. 5:**
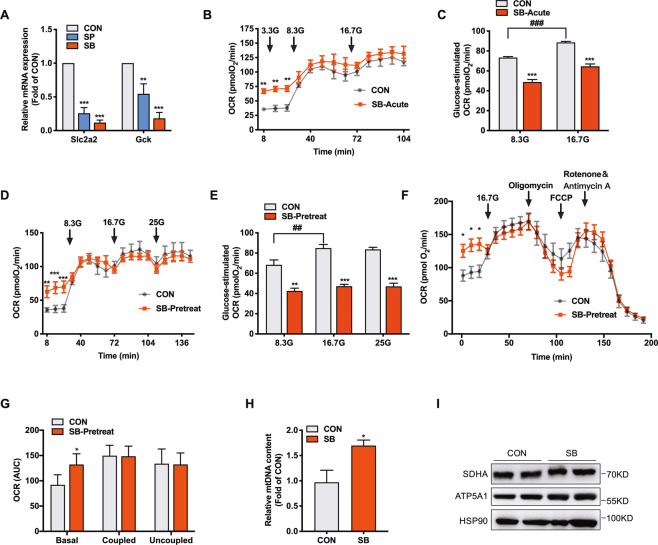
Sodium butyrate treatment decreases glucose oxidation in rat islets. **A** RT-qPCR analysis of Slc2a2 and Gck mRNA expressions in rat islets incubated with 5 mM sodium butyrate (SP) or 5 mM sodium propionate (SB) for 24 h. **B** Basal and glucose-stimulated oxygen consumption rate (OCR) in rat islets treated with 5 mM SB for 1 h. **C** Determination of glucose-stimulated OCR based on **B**. **D** Basal and glucose-stimulated OCR in rat islets treated with 5 mM SB for 24 h. **E** Determination of glucose-stimulated OCR based on D. **F** After rat islets were treated with 5 mM SB for 24 h, OCR was measured in the presence of 25 mM glucose, 1 μM oligomycin, 2 μM FCCP, or 0.5 μM rotenone/antimycin A at the indicated time points (arrows). **G** Determination of basal, coupled, and uncoupled respiration in **F**. **H** mtDNA content in INS-1 cells treated with 5 mM SB for 24 h. **I** SDHA (Complex II) and ATP5A1 (Complex V) protein expressions in INS-1 cells treated with 5 mM SB for 24 h. Data were given as mean ± SD for three separate experiments. *p < 0.05, **p < 0.01, ***p < 0.001 *vs* control (CON) group. ##*p* < 0.01, ###*p* < 0.001.

GSIS is usually linked to increased glucose metabolism through glycolysis and oxidation to generate ATP. The glucose flux into β cell is mainly determined by glucose transporter, Glut2 (encoded by Slc2a2) and glucokinase (encoded by Gck) [1]. Interestingly, Slc2a2 and Gck mRNA expressions exhibited a significant decrease in rat islets treated with SB or SP (Fig. 5A), contrary to the potentiated GSIS (Fig. 1A). We further measured oxygen consumption rate (OCR) in rat islets in response to glucose as an indicator of glucose oxidation with Seahorse XF24 analyzers. Basal OCR in rat islets incubated with 3.3 mM glucose was elevated by acute SB treatment. However, with elevated concentration of glucose, the difference of OCR between SB-treated and control islets disappeared (Fig. 5B). Glucose-stimulated OCR was significantly decreased in SB-treated rat islets compared with control islets (Fig. 5C). This was the case in rat islets pretreated with SB for 24 h (Fig. 5D, E). To further investigate the effect of SB on mitochondria respiration, we performed mitochondria stress assay in rat islets as shown in Fig. 5F. The initial OCR was evaluated to establish the basal respiration, the ATP-coupled OCR was determined following injection of the complex V inhibitor oligomycin, and the maximal respiration was evaluated upon addition of the uncoupling agent FCCP. SB pretreatment elevated mitochondrial basal OCR, but without effect on ATP-coupled and maximal respiration rates (Fig. 5G). The abundance of mitochondrial DNA was increased by SB (Fig. 5H). No obvious changes were observed in the levels of mitochondrial proteins SDHA and ATP5A1 between SB-treated and control islets (Fig. 5I). The mRNA levels of genes encoding enzymes critical for glycolysis, tricarboxylic acid (TCA) cycle, and oxidative phosphorylation showed no significant changes in SB-treated islets (Fig. S4A, B). These results indicate that SB-potentiated insulin secretion is independent of glucose oxidation.

### SB inhibits the expression of K_ATP_ and increases [Ca^2+^]_i_ in islet β cells

**Fig. 6:**
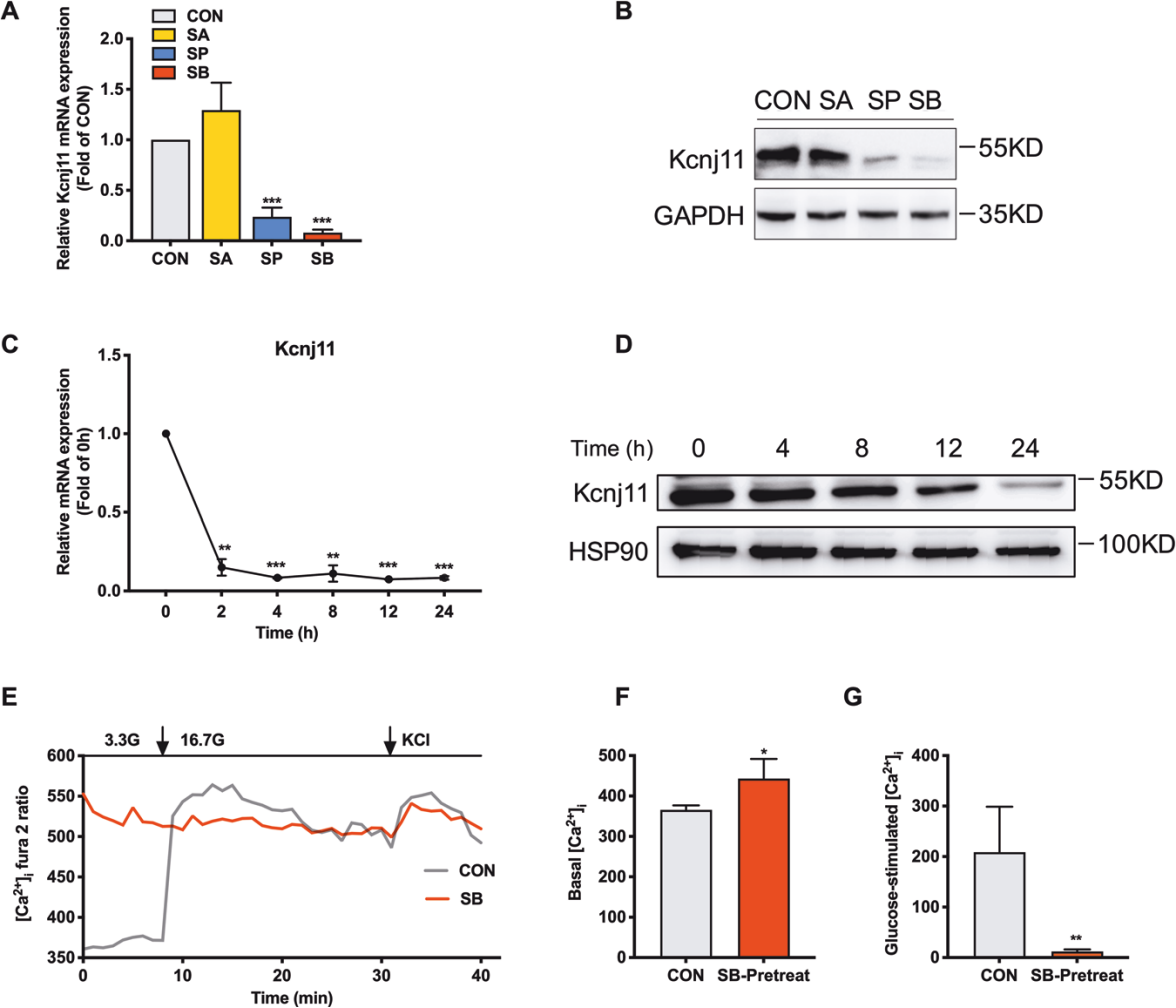
Sodium butyrate inhibits the expression of Kcnj11 and increases [Ca^2+^]_i_ in rat islet β cells. **A**, **B** Kcnj11 mRNA and protein expressions in rat islets treated with 5 mM sodium acetate (SA), 5 mM sodium propionate (SP), and 5 mM sodium butyrate (SB) for 24 h. **C**, **D** Kcnj11 mRNA and protein expressions in INS-1 cells treated with 5 mM SB for the indicated time. **E** Dispersed rat islet cells were incubated with 5 mM SB for 24 h and intracellular Ca^2+^ concentration ([Ca^2+^]_i_) was measured by patch-clamp technique. **F** Determination of basal [Ca^2+^]_i_ in **E**. **G** The increase of glucose-stimulated [Ca^2+^]_i_ in **E**. Data were given as mean ± SD for three separate experiments. *p < 0.05, **p < 0.01, ***p < 0.001 *vs* basal or control (CON) group.

K_ATP_ channels are essential for the process of stimulus-secretion coupling. K_ATP_ channels in β cell are composed of pore-forming Kir6.2 (encoded by Kcnj11) and regulatory SUR subunit (encoded by Abcc8) [33]. RNA-seq data showed that SB suppressed Kcnj11 mRNA expression in rat islets. RT-qPCR further confirmed that both SB and SP, not SA obviously decreased Kcnj11 mRNA expression in INS-1 cells (Fig. 6A). Kcnj11 protein expression exhibited a similar result (Fig. 6B). Surprisingly, the mRNA level of Kcnj11 decreased by 90% just 2 h after SB treatment (Fig. 6C). Kcnj11 protein expression was gradually attenuated with the prolonged incubation of SB, with the most profound action at 24 h (Fig. 6D). Considering the link of K_ATP_ channels to intracellular calcium concentration, we measured [Ca^2+^]_i_ in SB-pretreated rat islet β cells using patch clamp. As expected, the level of [Ca^2+^]_i_ at 3.3 mM glucose was relatively low and was significantly elevated by 16.7 mM glucose in the control group. In SB-pretreated β cells, the level of [Ca^2+^]_i_ at 3.3 mM glucose was near to the level triggered by high glucose in control β cells, not further raised by the addition of glucose (Fig. 6E). The basal level of [Ca^2+^]_i_ in SB-pretreated β cells was markedly higher compared with control β cells while high glucose-elicited [Ca^2+^]_i_ was considerably lower (Fig. 6F, G). Apparently, the lack of K_ATP_ channels induced by SB treatment triggers continuous depolarization of the β cell membrane, leading to Ca^2+^ influx and exocytosis of insulin secretory granules.

### H3K18bu occupation in insulin gene promoter is involved in SB-stimulated insulin gene expression

**Fig. 7:**
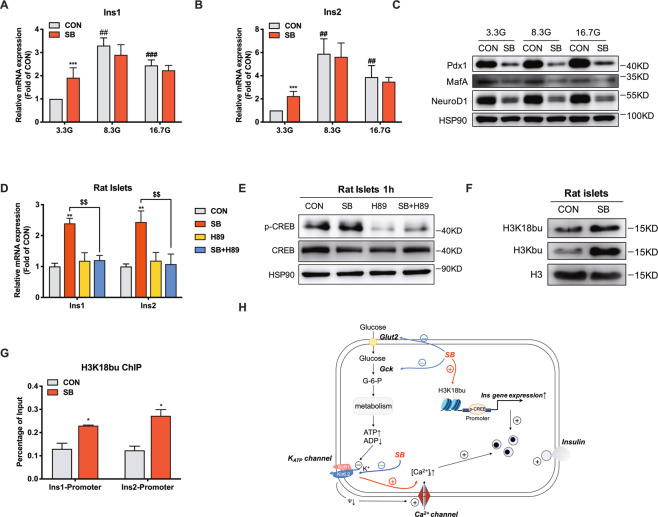
Sodium butyrate increases H3K18bu occupation in insulin gene promoter. **A**, **B** Ins1 and Ins2 mRNA expressions in rat islets treated with 5 mM sodium butyrate (SB) at 3.3, 8.3, or 16.7 mM glucose for 24 h measured by RT-qPCR. **C** Pdx1, MafA, and NeuroD1 protein expressions in rat islets treated with 5 mM SB at 3.3, 8.3, or 16.7 mM glucose for 24 h. **D** Ins1 and Ins2 mRNA expressions in rat islets incubated with 5 mM SB and 10 μM H89 for 24 h. **E** CREB phosphorylation level in rat islets incubated with 5 mM SB and 10 μM H89 for 1 h. **F** Global histone butylation and H3K18 butylation levels in rat islets treated with 5 mM SB for 8 h. **G** H3K18bu levels at the promoter region of Ins1 and Ins2 gene detected by ChIP-qPCR. **H** The schematic illustration summarizes the effect of SB on β cell function. Data were given as mean ± SD for three separate experiments. *p < 0.05, **p < 0.01, ***p < 0.001 *vs* control (CON). ##*p* < 0.01, ###*p* < 0.001 vs 3.3 mM glucose (3.3 G). $$*p* < 0.01.

In accordance with RNA-seq data, RT-qPCR validated the upregulation of Ins1 and Ins2 expression in rat islets treated with SB at 3.3 mM glucose, not at 8.3 and 16.7 mM glucose (Fig. 7A, B). It is widely recognized that the insulin gene expression is governed by three transcription factors Pdx1, MafA, and NeuroD1 [34–36]. However, SB significantly decreased the protein levels of the three key transcription factors (Fig. 7C), excluding their involvement in SB-induced insulin gene expression. It has been reported that rat insulin promoter contains an active cAMP-responsive element (CRE) [37]. Indeed, SB-induced Ins1 and Ins2 gene expressions were abolished by H89, a PKA inhibitor (Fig. 7D). However, the phosphorylation of CREB was not stimulated by SB (Fig. 7E). Recently, increasing evidence has shown that beyond acetylation, other lysine acylation modifications such as butyrylation and succinylation are involved in gene expression regulation [38, 39]. The substrates for these modifications, such as butyryl-CoA and succinyl-CoA are derived from metabolic intermediates of glucose, fatty acid, or other nutrients. SB could be metabolized to butyryl-CoA [40]. As expected, an obvious increase in total histone butyrylation level was observed in SB-treated INS-1 cells (Fig. 7F). Furthermore, ChIP-qPCR result showed that SB increased H3K18bu levels in the promoter regions of Ins1 and Ins2 (Fig. 7G). Therefore, it is likely that SB increases H3K18bu levels to promote chromosome opening, leading to an increase in insulin gene expression.

## Discussion

It is generally accepted that SCFAs generated by gut microbiota exert a beneficial impact on energy metabolism via central mechanisms and peripheral actions [18]. The role of SCFAs in islet function should be paid more attention as insulin is the central regulator of metabolism. However, there exist contradictory results about their action in this aspect [41]. The present study revealed a strong insulinotropic function of SCFAs after a long-term incubation. Surprisingly, SB treatment led to an unusual transcriptome change, with downregulation of multiple β cell identity genes. In addition, it seemed to be conflicting that enhanced GSIS was accompanied with reduced glucose-stimulated OCR in rat islets treated with SB. Our study further demonstrated that SB enhanced insulin gene expression through increasing H3K18bu occupation in its promoter and potentiated insulin secretion via elevating [Ca^2+^]_i_ mediated by the ablation of K_ATP_ channels, uncovering a novel mechanism underlying the potentiation of islet function independent of key β cell transcription factors and functional genes.

It has been well established that fatty acids are essential for insulin secretion. Acute lowering of plasma fatty acid level decreased the basal and glucose-induced insulin secretion [12, 42]. On the contrary, the elevation of fatty acid level significantly enhanced GSIS in vitro and in vivo [43, 44]. The potency of fatty acid to promote insulin secretion was subjected to chain length and degree of saturation [14]. Long-chain fatty acid palmitate and medium-chain FA octanoic acid potentiate acute insulin secretion at basal and high glucose concentrations [45, 46]. SCFAs acetate, propionate, and butyrate improve glucose tolerance, insulin sensitivity, and body weight [18]. The in vivo beneficial impact of SCFAs on β cell function is attributed to the elevation of GLP-1. However, the direct effect of SCFAs on insulin secretion remains controversial [47, 48]. There are reports that SCFAs exhibit inhibitory, stimulating, or no acute effect on insulin secretion [41]. Little is known about the long-term impact of SCFAs on islet β cell function. In this current study, none of the three SCFAs displayed acute effects on basal and glucose-induced insulin secretion. However, the pretreatment of SA, SP or SB for 24 h significantly amplified insulin secretion from isolated rat islets in response to various stimuli. Moreover, the insulinotropic effect increased with the chain length, with SB showing the most profound effect.

Histone acetylation loosens the chromatin structure and promotes gene transcription whereas histone deacetylation exerts a contrary action. It has been reported that HDACs are expressed in the pancreas [49] and are involved in the development, differentiation, proliferation, and function of islet β cells [50]. H3K27ac was previously revealed to be associated with both active promoters and enhancers in human islet cells [51] and in mouse islets [52]. In the present study, the analysis of RNA-seq and H3K27Ac ChIP-seq supported that SB-induced transcriptome change was partially dependent on its role as an HDAC inhibitor. Several genes involved in the metabolism pathway, such as Aldh2 and Acadm were upregulated by SB as their promoter regions displayed hyperacetylation of H3K27. Unusually, SB selectively inhibited the expressions of multiple β cell identity genes. However, their expressions barely showed any changes in TSA-treated islets. Nor did the H3K27Ac peaks in the promoter of these genes in SB-treated islets. These results suggest that the inhibitory effect of SB on β cell identity genes is independent of its deacetylation action.

The magnitude of GSIS is tightly linked to the rate of glucose metabolism mediated by the permissive rates of glucose uptake and phosphorylation. Glucose transport into β cell is mediated by transporters Glut1 in humans or Glut2 in rodents [1]. Following the entry into the β cell, glucose is rapidly phosphorylated by Gck, a rate-limiting enzyme. and then metabolized through glycolytic and oxidative pathways. Glut2 and Gck have been considered as the “glucose sensors” in β cells [53]. Mutations in the human GCK gene lead to monogenic diabetes (MODY2) [54]. In this current study, Slc2a2 and Gck mRNA expressions were significantly attenuated in INS-1 cells and rat islets in exposure to SB. Considering the central role of Glut2 and Gck in glucose metabolism, it is reasonable to suppose that the glucose flux into β cell is decreased in SB-treated islets. As expected, glucose-stimulated oxidation was significantly reduced by SB treatment, but the genes involved in glycolysis and TCA barely showed any changes. Moreover, no obvious alterations in mitochondrial function were observed in SB-treated rat islets. These results indicate that the reduction of glucose oxidation induced by SB is due to the decreased glucose flux into β cells. It seems strange that glucose-induced insulin secretion is decoupled with its metabolism in rat islets treated with SB. Apparently, SB-repressed Kcnj11 expression and subsequent increased [Ca^2+^]_i_ could partially address its insulinotropic action.

The sustained proinsulin biosynthesis ensures sufficient insulin content for secretion, which is tightly regulated at the transcription level. Glucose is a key regulator of insulin gene transcription [55]. It has been demonstrated that three β cell-specific transcription factors Pdx1, NeuroD1, and MafA play a crucial role in glucose-regulated insulin gene transcription and β cell function [34–36]. Moreover, mutations in the human Pdx1 and NeuroD1 genes lead to MODY4 and MODY6, respectively. In addition, there also exists a CRE in the promoter of rat Ins1 and Ins2 genes [56]. cAMP analogs have been shown to increase insulin mRNA levels in rat and human islets via stimulating CREB phosphorylation and its binding with CRE in the promoter of the insulin gene. Deletion or mutagenesis of this sequence abolished the cAMP stimulatory effect [56]. In addition to transcription factors, an open chromosome structure mediated by histone modification is also indispensable to induce transcriptional activation. It was reported that high glucose promoted histone acetylation in the promoter of the insulin gene and elicited insulin gene transcription in MIN-6 cells [57]. SB increased insulin gene expression and β cell function in the islets of juvenile diabetic rats [58] and the rat insulinoma β cell line RIN 1046-38 [59]. In our study, the mRNA levels of rat Ins1 and Ins2 genes were increased in rat islets treated with SB while Pdx1, MafA, and NeuroD1 displayed significant decreases at mRNA and protein levels. The phosphorylation of CREB was not stimulated by SB. Additionally, unlike SB, TSA did not induce insulin gene expression, indicating involvement of other mechanisms in SB-triggered insulin transcription. Besides acetylation, histone butyrylation could also directly stimulate gene transcriptional activity [60]. In the liver, H3K18bu was downregulated by a high-fat diet [61]. In our study, SB treatment increased the H3K18bu level in the promoters of Ins1 and Ins2 genes. It is likely that SB stimulates insulin gene transcription through increasing the H3K18bu level.

In summary, it is an interesting finding that SB treatment enhances insulin secretion from rat islets while downregulating a set of β cell identity genes. Apparently, SB-repressed β cell key functional genes and transcription factors are independent of its deacetylase activity. SB-potentiated β cell function is attributed to the elevated [Ca^2+^]_i_ via decreasing K_ATP_ channels and increased insulin gene transcription due to H3K27bu occupation in its promoter, independent of glucose oxidation. Our work indicates a unique mechanism by which SB potentiates insulin secretion at the expense of compromised islet β cell identity gene expressions.

## Methods

### Islet isolation, cell culture, and treatment

Pancreatic islets were isolated from 8- to 10-week-old wild-type Sprague-Dawley (SD) male rats by collagenase digestion and density-gradient centrifugation. INS-1 cells were cultured in RPMI 1640 medium with 11.1 mM glucose that contained 10% FBS, 10 mM HEPES, 1% penicillin/streptomycin, 1 mM sodium pyruvate, and 50 μM beta-mercaptoethanol. Plasmid transfection was performed with Lipofectamine 3000 (Invitrogen). SA, SP, and SB were purchased from Sigma-Aldrich.

### Insulin secretion assay

Isolated islets were pre-incubated in Krebs-Ringer Buffer (KRB) containing 3.3 mM glucose at 37 °C for 30 min. Ten islets per assay in triplicate were then incubated with KRB buffer containing other additions as indicated for 1 h at 37 °C. The supernatant was collected for insulin secretion analysis, and islets were then extracted with acid-ethanol to determine total insulin content. Insulin levels were estimated using an ELISA kit (Mercodia, St Charles, MO).

### RNA sequencing analysis

Isolated rat islets were cultured in RPMI 1640 medium with 3.3 mM glucose and 0.25% BSA in the presence or absence of 5 mM SB for 24 h. 1 μg total RNA was used to prepare RNA-seq libraries by the KAPA Stranded RNA-Seq Library Prep Kit (Illumina), and then deep sequencing was performed with an Illumina HiSeq 4000 by KangChen Biotech Company (Shanghai, China). RNA-seq reads were compared to Rattus norvegicus genome. The differentially expressed genes and transcripts were identified by setting a threshold at fold change > 2.0, *p*-value < 0.001.

### ChIP-seq

Isolated rat islets incubated with or without 5 mM SB for 24 h were crosslinked with 1% formaldehyde, lysed, and sonicated to shear the chromatin into appropriate fragments. Immunoprecipitation was performed with the H3K27Ac antibody (Millipore). TruSeq Nano DNA Sample Prep Kit (Illumina) was used for sequencing library preparation. Sequencing was performed on Illumina HiSeq 4000 using HiSeq 3000/4000 SBS Kit for 300 cycles. MACS v1.4.2 (Model-based Analysis of ChIP-seq) software was run with the mapped reads to detect the statistically significant ChIP-enriched peaks compared with the respective input group by a *p*-value threshold of 10^−4^. All regions were annotated by the gene whose TSS was nearest to the center of the peak region according to the newest UCSC RefSeq database and divided into five classes based on the distance to UCSC RefSeq genes. Coverage, reads, and peaks were visualized with Integrative Genomics Viewer (IGV).

### Microarray expression analysis

Total RNA was extracted from isolated rat islets treated with or without 200 nM TSA for 24 h using Trizol (Invitrogen). Sample labeling and array hybridization were performed according to the instructions for the Agilent One-Color Microarray-Based Gene Expression Analysis protocol (Agilent Technologies, Inc., Santa Clara, CA, USA). Labeled cRNAs were hybridized onto the Whole Genome Oligo Array (4 × 44 K; Agilent Technologies) and the arrays were scanned by the Agilent Scanner G2505C. Agilent Feature Extraction software (version 11.0.1.1) was used to analyze acquired array images.

### RNA isolation and RT-qPCR

Total RNA was extracted using RNeasy Plus Mini Kit (including a DNase digestion step, Qiagen GmbH, Germany) from islets and the first strand of cDNA was synthesized using the reverse transcription kit (Toyobo Co., Ltd., Osaka, Japan). RT-qPCR was performed using Applied Biosystems 7300 Real-Time PCR machine (Applied Biosystems, Foster City, CA, USA) and the reagent SYBR Premix Ex Taq II (Takara, Shiga, Japan). The relative mRNA expression level of each gene was normalized to that of 18 S or β-actin. The primer sequences were described in Supplemental Table 1.

### Western blot

Isolated rat islets or INS-1 cells were treated with RIPA lysis buffer for total protein extraction. Blotted membrane was imaged with a LAS-4000 Super CCD remote control science imaging system (Fuji). Antibody information was listed in Supplemental Table 2.

### Chromatin immunoprecipitation (ChIP)

The ChIP assay was performed using an EZ-ChIP Chromatin Immunoprecipitation kit (EMD Millipore, Billerica, MA, USA) according to the manufacturer’s protocol. In brief, rat islet cells were fixed using the formaldehyde with 1% formaldehyde and homogenized in cell lysis buffer. DNA was sheared to fragments at 200–1000 bp using sonication. Chromatins were incubated and precipitated with antibody against H3K18bu or IgG (both from PTM Biolabs). DNA pellets were analyzed by real-time qPCR by using primers directed to the Ins1 promoter and Ins2 promoter (Supplemental Table 1).

### [Ca^2+^]_i_ recording in single islet β cells

[Ca^2+^]_i_ was measured by placing the coverslip on the stage of IX71 inverted microscope (Olympus). The single islet cells were loaded with 4 µM Fura 2-AM (Sigma) for 30 min at 37 °C in KRBB containing 3 mM glucose, then washed with KRBB, and stimulated with 16.7 mM glucose or 35 mM KCl. The single cells were illuminated by excitation at 340 nm (F340) and 380 nm (F380) using a monochromator (Till Photonics, Munich, Germany), and the emission signals were detected at 510 nm using an image-intensifying CCD camera (SensiCam, PCO, Kelheim, Germany). The images were collected at 10 s intervals. The [Ca^2+^]_i_ was expressed as the ratio of F340/F380 (AU).

### Oxygen consumption rate measurement

Isolated rat islets were pretreated with 5 mM SB for 24 h, then the oxygen consumption rate of isolated islets was measured using a Seahorse XF24 flux analyzer (Seahorse Bioscience) according to the manufacturer’s instructions. In brief, for glucose oxidation assay, 8.3, 16.7, or 25 mM glucose was added to stimulate cellular oxygen consumption. For the mitochondrial stress test, 1 μM oligomycin, 2 μM FCCP, and 0.5 μM rotenone/antimycin A were added at the indicated time.

### Statistics

Data were presented as means ± SD. Comparisons were performed by Student’s *t*-test for two groups or *ANOVA* for multiple groups. Significance was established at *p* < 0.05.

## Supplementary information

Supplementary information includes 4 figures and 2 tables.

Reproducibility Checklist

## Data Availability

The data sets generated during the current study are available from the corresponding author on reasonable request.
