# Supplementary material for: Sodium butyrate potentiates insulin secretion from rat islets at the expense of compromised expression of β cell identity genes
Source: Cell Death Dis. 2022 Jan 19;13(1):67. doi: 10.1038/s41419-022-04517-1 (PMC8770496; doi:10.1038/s41419-022-04517-1)
Supplement: Supplementary file 1 — Supplementary information includes 4 figures and 2 tables. [file 41419_2022_4517_MOESM1_ESM.pdf]

Supplementary information includes 4 figures and 2 tables.

Supplementary Figure 1. The comprehensive impacts of short-chain fatty acids on insulin secretion.

(A) Rat islets were stimulated with 3.3, 8.3 or 16.7 mM glucose in the presence or absence of 5 mM sodium acetate (SA), 5 mM sodium propionate (SP) or 5 mM sodium butyrate (SB) for 1 h, and insulin secretion was measured. (B) Insulin content of rat islets pretreated with 5 mM SA, 5 mM SP or 5 mM SB for 24 h and stimulated with 3.3, 8.3 or 16.7 mM glucose for 1 h. (C) INS-1 cells were incubated with 5 mM SA, 5 mM SP or 5 mM SB for 24 h, and then cell viability was detected using CCK8 assay. (D) Rat islets were stimulated with 10 μM forskolin (FSK), 5 mM glutamine (Gln) and leucine (Leu), or 35 mM KCl in the presence or absence of 5 mM SB for 1 h, and insulin secretion was measured. Data were given as mean ± SD for three separate experiments.

Fig.S1

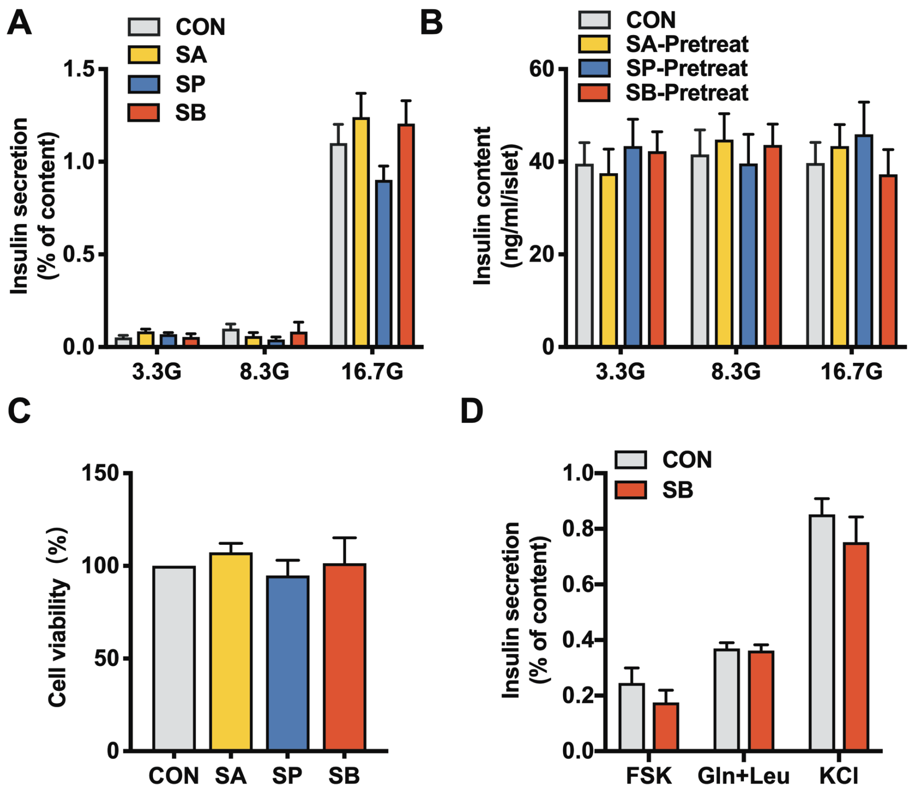

Supplementary Figure 2. The profound effects of various agents on β cell gene expressions.

(A) Expressions of β cell identity genes in mouse islets incubated with 5 mM sodium butyrate (SB) for 24 h detected by RT-qPCR. (B-C) After INS-1 cells and mouse islets were treated with 5 mM SB for 24 h and then remained in SB-free medium for another 24 h, gene expressions were detected. (D) DNMT3A protein expression in rat islets incubated with 5 mM SB for 24 h. (E) Acetylated histone H3, H2, and total histone H3 levels in INS-1 cells treated with 5 mM SA, 5 mM SP, 5 mM SB, 3 μM MS, and 200 nM TSA. Data were given as mean ± SD for three separate experiments. $^{*}p < 0.05$, $^{**}p < 0.01$, $^{***}p < 0.001$ *vs* control (CON) group; $^{\#}p < 0.05$, $^{\#\#}p < 0.01$, $^{\#\#\#}p < 0.001$ *vs* 24h treatment group.

Fig.S2

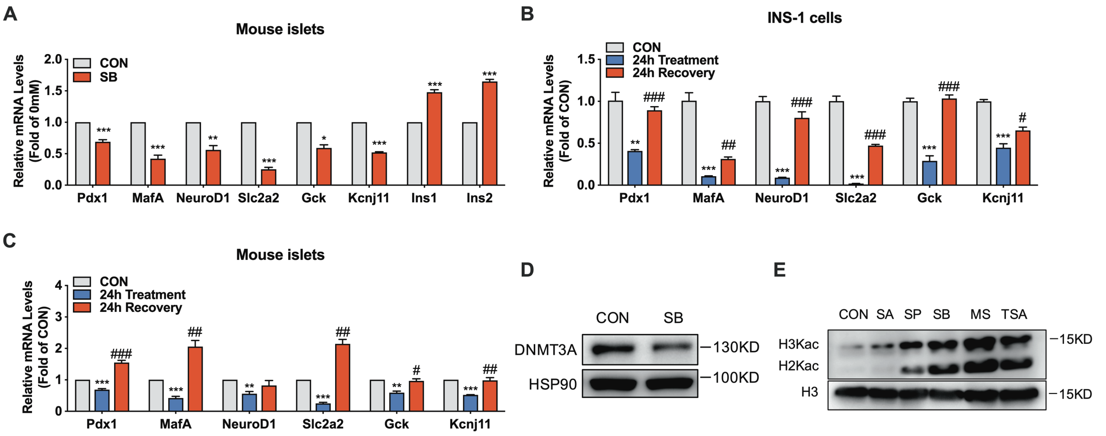

Supplementary Figure 3. Effects of sodium butyrate and trichostatin A on the expression of islet β cell identity genes.

The expressions of multiple islet β cell identity genes were compared from two sets of gene expression profiles of sodium butyrate (SB)- and trichostatin A (TSA)-treated rat islets.

Fig.S3

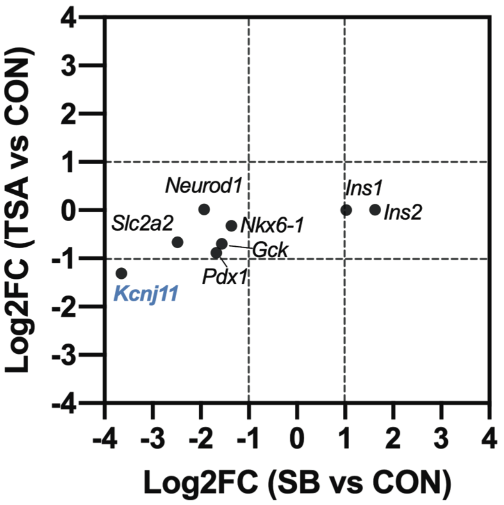

Supplementary Figure 4. Effects of sodium butyrate on the expressions of genes involved in glycolysis or mitochondrial function.

(A) The gene expression pattern of glycolysis pathway was graphically represented: green indicates downregulation (fold change ≤0.5), orange indicates upregulation (fold change ≥2), and blue indicates non-significant changes. (B) Sodium butyrate had no significant impacts on the expressions of mitochondrial function-related genes.

Fig.S4

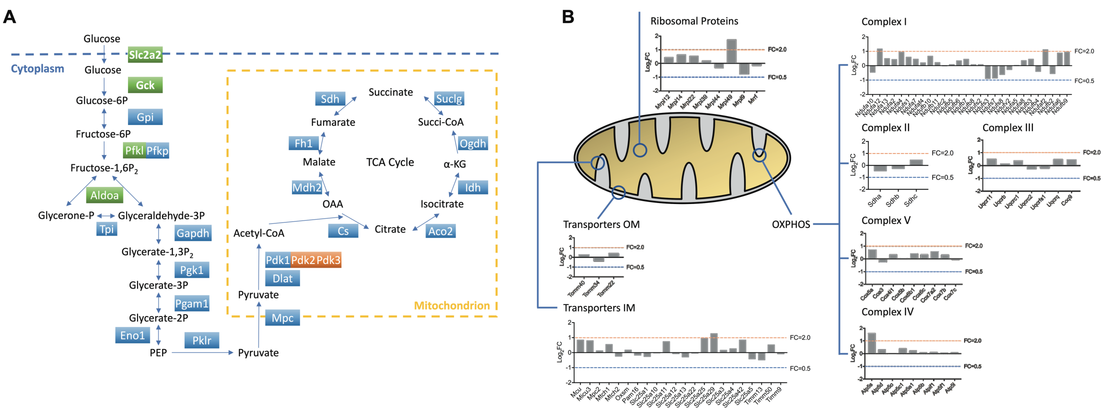

Supplementary Table 1. Sequences of primers for realtime-PCR and ChIP-PCR

**Sequences of primers for realtime-PCR**			
Gene Name	Species	Primer sequence (Forward: 5’-3’)	Primer sequence (Reverse: 5’-3’)
18S	Rat	CACGGGTGACGGGGAATCAG	CGGGTCGGGAGTGGGTAATTTG
Pdx1	Rat	CTTTCCCGAATGGAACCGAG	GAATTCCTTCTCCAGCTCC
MafA	Rat	AGCAAGGAGGAGGTCATC	CGTATTTCTCCTTGTACAGG
Nkx2.2	Rat	GACCTTCCGGACACCAACGA	AGCGAGTGTAGGGGTTGTCG
Nkx6.1	Rat	GAGAGAGCACGCTTGGCCTA	GGGTCCAGGGGCTTGTTGTA
NeuroD1	Rat	GGCTCCAGGGTTATGAGATC	GCATTCATGGCTTCAAGC
Slc2a2	Rat	TCAGCCAGCCTGTGTATGCA	TCCACAAGCAGCACAGAGACA
Gck	Rat	AGTGGCCACAATGATCTCCTG	TTCCACCAGCTCCACATTCTG
Kcnj11	Rat	ACTTTGTCTGGGGGACACTG	TGGGAGGCTTTATTGACGAC
Ins1	Rat	CCTGCTCGTCCTCTGGGAGCCCAAG	CTCCAGTGCCAAGGTCTGAAGATCC
Ins2	Rat	CCTGCTCATCCTCTGGGAGCCCCGC	CTCCAGTGCCAAGGTCTGAAGGTCA
Hnf1α	Rat	CAACCACCCTCTCTCCCAGTAAG	GACACTGTGACTAAGGGACCTCC
Dnmt3a	Rat	GGATGATCGAAAGGAAGGAGAGG	TCCGCTTCTCCAAGTCTCCA
Aldh2	Rat	GGCTGTTGTACCGATTGGCT	CCAGCCAGCATAATAGCGGAG
Acadm	Rat	AGCTGATGATGTGTGCCTACTG	CCACGATGAATCCGGTGAAGG
Gcdh	Rat	CCACGATGAATCCGGTGAAGG	GGGAGTTGGTGATCCAGGTCTT
Tph1	Rat	TGCGACATCAACCGAGAA	GCAGAAGTCCAGGTCAGAAAT
Dis3l	Rat	GTGCGCGAACACTATCTGC	CAGTTTGTTATACTGTCTCCGGC
Zbed3	Rat	CAAAATGTCTGCGAGGAGCC	CAGGTGAAAGTAGCCCCAGG
Aars	Rat	GATTGGGTGGTGAGGTACGG	AGGAAGATGGGTTTGAACTGGT
β-actin	Mouse	GTGACGTTGACATCCGTAAAGA	GTGACGTTGACATCCGTAAAGA
Pdx1	Mouse	TTCCCGAATGGAACCGAGCCTG	TTTTCCTCGGGTTCCGCTGTGT
MafA	Mouse	TTCAGCAAGGAGGAGGTCAT	CTCTGGAGCTGGCACTTCTC
NeuroD1	Mouse	AGGAATTCGCCCACGCAGAA	GGTCATGTTTCCACTTCCTGTTGT
Slc2a2	Mouse	TCATGTCGGTGGGACTTGTG	CCCAAGGAAGTCCGCAATGT
Gck	Mouse	TGACTCTGTGGGGGAAGTCT	GCCAGGATCTGCTCTACCTTT
Kcnj11	Mouse	TGGGGGCTCAGTAAGCAATG	AGCCGGGTCAGCACATATTC
Ins1	Mouse	TAGTGACCAGCTATAATCAGAG	ACGCCAAGGTCTGAAGGTCC
Ins2	Mouse	GCAAGCAGGAAGGTTATTGTTTCAA	GCTCCAGTTGTGCCACTTGT
**Sequences of primers for ChIP-PCR**			
Location	Species	Primer sequence (Forward: 5' -3')	Primer sequence (Reverse: 5' -3')
Ins1 promoter	Rat	TGAACGCTGTGCTACTGAGG	CCACAAACCCATAGCCCACT
Ins2 promoter	Rat	GAGGACACAGCTATCAGTGGG	CACATCATTCCCCAGGAGGC

Supplementary Table 2. Antibodies used in this article.

**Antibodies**	**Vendor**	**Catalog number**
Rabbit Anti-Pdx1	CST	Cat.# 5679
Rabbit Anti-SDHA	CST	Cat.# 11998
Rabbit Anti-ATP5A1	Abcam	Cat.# ab176569
Rabbit Anti-Hsp90	Millipore	Cat.# 07-2174
Rabbit Anti-MafA	Bethyl	Cat.# A700-067
Rabbit Anti-Nkx6.1	Abcam	Cat.# ab221549
Mouse Anti-Kac	CST	Cat.# 9681
Rabbit Anti-Dnmt3a	CST	Cat.# 32578
Mouse Anti-KIR6.2	Santa Cruz Biotechnology	Cat.# sc-390104
Rabbit Anti-CREB	CST	Cat.# 9197
Rabbit Anti-P-CREB(Ser133)	CST	Cat.# 9198
Rabbit Anti-Kbu	PTM BIO	Cat.# PTM-301RM
Rabbit Anti-H3K18bu	PTM BIO	Cat.# PTM-306
HRP-linked Anti-Rabbit	CST	Cat.# 7074
HRP-linked Anti-Mouse	CST	Cat.# 7076
